# Supplementary material for: Amikacin liposome and Mycobacterium avium complex: A systematic review
Source: PLoS One. 2022 Dec 27;17(12):e0279714. doi: 10.1371/journal.pone.0279714 (PMC9794038; doi:10.1371/journal.pone.0279714)
Supplement: Attachment — Submitted filename: Response to Reviewers.docx [file pone.0279714.s003.docx]

Dear Editor

We thank the editor and the reviewers for their comments on our manuscript. Below is our response to each point raised by the academic editor and reviewers. We hope that we satisfyingly addressed them and that the manuscript will be now suited for publication.

Sincerely,

On behalf of all authors,

**Mohammad Javad Nasiri**

**Response to Editor comments:**

Thank you for considering our manuscript. We thank the editor and reviewers for their thoughtful critique and comments. We have carefully edited the manuscript as requested by you and have provided a point-by-point response below. Please find the revised version included. The revisions are highlighted in yellow in the resubmitted manuscript. We hope this meets the established reputation for the quality of your esteemed journal.

1) Thanks for pointing this out. The manuscript is updated with PLOS ONE's style requirements.

2) Thanks for your fair and constructive comment. The duplicated text is rephrased word by word.

3) Thanks for pointing this out. All your queries about financial disclosure are addressed in cover letter and Funding Statement section of the online submission form

4) Thanks for pointing this out. The acknowledgement is corrected (Page 13, line: 267) and funding information is addressed in cover letter and Funding Statement section of the online submission form.

5) Thanks for pointing this out. The data availability statement and captions for Supporting Information files (search strategy) are added. (Page 13, lines: 261-263)

**Response to reviewer 1**

We appreciate the time and attention you spent in reviewing our manuscript and your thoughtful critique and comment.

Brief descriptions of some clinical cohorts and systematic reviews about safety and efficacy of injectable amikacin are added to the discussion section of manuscript and highlighted in yellow, for comparing the benefits of LAI against conventional routes of amikacin delivery. (Pages 10 & 11, lines: 213-224)

**Response to reviewer 2**

Thank you for your willingness to consider our initial manuscript “Amikacin liposome and Mycobacterium avium complex: A systematic review”. We have carefully considered all comments and revised and improved some parts of the original manuscript as requested. We hope this meets the established reputation for the quality of your esteemed journal. The revisions are highlighted in yellow in the resubmitted manuscript.

1) Thanks for your fair and constructive comment. The simplification has been done. (Page 2, lines: 43-45)

2) Thanks for pointing this out. The phrase that you are looking for is added. (Page 2, lines: 45 and 46)

3) Thanks for your recommendation. The term "sustainable" has been changed to "sustained". Sustained and durable culture conversion is defined by negative sputum culture results for 12 months during treatment and for 3 months after treatment respectively. (Page 2, lines: 52-54)

4) Thanks for your suggestion. The basic information of NTM is added. (Page 3, lines: 63 and 64)

5) Thanks for your recommendation. The description (chronic obstructive pulmonary disease (COPD), bronchiectasis, and cystic fibrosis) is added to the text. (Page 3, lines: 66-68)

6) Thanks for your suggestion. The phrase has been revised. (Page 3, line: 70)

7) Thanks for pointing this out. The phrase that you are looking for is added. (Page 10, line: 207)

8) Thanks for your suggestion. This sentence has been clarified (advanced or previously treated MAC lung disease). (Page 10, lines: 209-212)

9) Thanks for your recommendation. Some of the mentioned sentences have been deleted and some parts have been revised. (Page 11, Lines: 225-227)

10) Thanks for your fair and constructive comment. To clarify the sentence, it is changed to “Likewise, inhaled antibiotic can be effective and practically feasible in cases of M. tuberculosis infection”. (Page 11, lines: 234-235)

11) Thanks for pointing this out. The phrase that you are looking for is added. (Page 11, lines: 237 and 238)

12) Thanks for pointing this out. The acknowledgement has been corrected in order to journal requirements. And your revised phrase is added in financial information section in online submission form. (Page 13, line: 267)
